# Poetry

**Published:** 1879-10

**Authors:** 


### POETRY.

THAT good poetry, whether in rhyme or blank verse, has a power over the mind to raise it to the highest pitch of enthusiasm, or even frenzy, or to calm it; like oil thrown on troubled waters, when it has been carried away by boisterous passion, or almost crazed with overwhelming troubles, can not be denied. History, as well as individual experience, strongly confirms the fact. An old familiar hymn, even the sudden remembrance of doggerel rhymes which were learned in innocent childhood, sometimes break over the memory with resistless power, when half a century has passed away, and act on the mind as anodynes upon the physical system. There are times to all when it is profitable to hie away to some solitary nook, when the spirit is in the minor mood or in a perturbed condition, and read with deliberation what some wayfarer, who has gone before, has penned to calm, it may be, his own sorrow or sadness.

